# Drug-Induced Autoimmune Hepatitis: An Unusual Adverse Event of Atorvastatin Therapy

**DOI:** 10.7759/cureus.55809

**Published:** 2024-03-08

**Authors:** Mitwa Patel, Abdul Fattah, Helai Hussaini, Fnu Maneesha, Zahoor Ahmed

**Affiliations:** 1 Internal Medicine, David Tvildiani Medical University, Tbilisi, GEO; 2 Internal Medicine, Liaquat University of Medical and Health Sciences, Jamshoro, PAK; 3 Internal Medicine, West Anaheim Medical Center, Anaheim, USA; 4 Internal Medicine, Ghulam Mohammad Mahar Medical College, Sukkur, PAK; 5 Internal Medicine, Mayo Hospital, Lahore, PAK

**Keywords:** drug induced hepatitis, drug induced liver injury, autoimmune hepatitis, atrovastatin induced hepatitis, statin induced hepatitis

## Abstract

Drug-induced autoimmune hepatitis (AIH) is characterized by acute or chronic hepatic injury coupled with autoantibody development, hypertransaminasemia, and idiopathic AIH features on liver biopsy. Atorvastatin-induced AIH is a rare but well-documented life-threatening adverse event. We report a case of atorvastatin-induced AIH in a 57-year-old female who presented with worsening fatigue, jaundice, and deranged liver function tests. She had been prescribed atorvastatin 20 mg daily three months prior. Her clinical presentation, imaging findings, and serological testing were suggestive of drug-induced AIH. A liver biopsy confirmed a drug-induced autoimmune picture, and she was diagnosed with atorvastatin-induced AIH after ruling out all other possible causes. Her clinical presentation and liver enzymes improved after prolonged treatment with prednisone.

## Introduction

Drug-induced liver injury (DILI) with autoimmune features, also called drug-induced autoimmune hepatitis (AIH), is characterized by acute or chronic hepatic injury coupled with autoantibody development, a hepatocellular pattern of serum enzyme elevations, and idiopathic AIH features on liver biopsy [1]. Drug-induced AIH shares clinical and pathological characteristics similar to idiopathic AIH, including serological features and histological characteristics [2]. Drug-induced AIH is a rare but documented cause of liver failure and transplantation. The incidence of drug-induced AIH constitutes approximately 2%-18% of AIH and accounts for 2.9%-8.8% of DILI [3]. Various drugs have been implicated in drug-induced AIH, including hydralazine, minocycline, methyldopa, fenofibrate, and nitrofurantoin [4]. Statins are also well-documented but rare etiologies of drug-induced AIH. Drug-induced AIH induced by atorvastatin is rarely reported in the literature [5]. We report a case of drug-induced AIH induced by atorvastatin.

## Case presentation

A 57-year-old female with a history of type 2 diabetes mellitus (DM) and dyslipidemia presented with worsening fatigue and generalized pruritus for the last month. She also reported a yellowish discoloration of her eyes and non-documented weight loss. She was diagnosed with type 2 DM seventeen years ago and was switched to insulin therapy one year ago due to poor control. She was diagnosed with dyslipidemia two years ago and was prescribed atorvastatin 20 mg daily three months ago after failing to reduce her weight through lifestyle and dietary modifications. She had no history of autoimmune or liver disease and reported no history of alcohol abuse, smoking, substance abuse, or recent travel. She had no family history of a similar disease. She reported no history of any herbal medication use.

On examination, she was hemodynamically stable. Icteric sclera and generalized jaundice were noted, and an abdominal examination revealed mild tenderness in the right upper quadrant. The rest of the systemic examination was unremarkable. Her initial laboratory evaluations were within normal range except for elevated transaminases (Table 1). Her liver and renal function tests were within normal range three months ago. Abdominal ultrasound revealed homogeneous parenchymal echogenicity.

**Table 1:** Initial results of laboratory evaluations.

Parameter	Lab results	Reference value
Alanine aminotransferase	251	0-55 U/L
Alkaline phosphatase	391	40-150 U/L
Aspartate aminotransferase	445	5-34 U/L
Gamma-glutamyl transferase	588	12-64 U/L
Total bilirubin	6.9	0.2-1.2 mg/dL

Atorvastatin was discontinued, and she was managed with symptomatic treatment. Her liver function tests remained elevated after two weeks. She underwent a detailed serological workup; the results are shown in Table 2. Her urine drug screening was negative, and serological testing was negative for hepatitis A, B, and C. Her serology testing was also negative for human immunodeficiency virus, cytomegalovirus, and Epstein-Barr virus. Her antinuclear antibody titer was at 1:180; however, serum immunoglobulin (Ig) G level was within normal range, and smooth muscle antibodies, anti-mitochondrial antibodies, and liver-kidney microsomal antibodies were negative. Magnetic resonance cholangiopancreatography was normal without biliary stricture or stones.

**Table 2:** Results of a detailed workup.

Parameter	Lab results	Reference value
Alanine aminotransferase	201	0-55 U/L
Alkaline phosphatase	332	40-150 U/L
Aspartate aminotransferase	415	5-34 U/L
Gamma-glutamyl transferase	504	12-64 U/L
Total bilirubin	6.9	0.2-1.2 mg/dL
Direct bilirubin	5.2	0-0.5 mg/dL
Prothrombin time	16	10-13 seconds
International normalized ratio	1.2	1.0
Fibrinogen	162	200-400 mg/dL
Total protein	4.9	6-8.3 g/dL
Serum albumin	3.3	3.4-5.4 g/dL

Based on serological tests and imaging results, her clinical picture favored an adverse reaction to atorvastatin, and she was commenced on oral prednisone 60 mg daily with a plan of subsequent taper.

**Figure 1:**
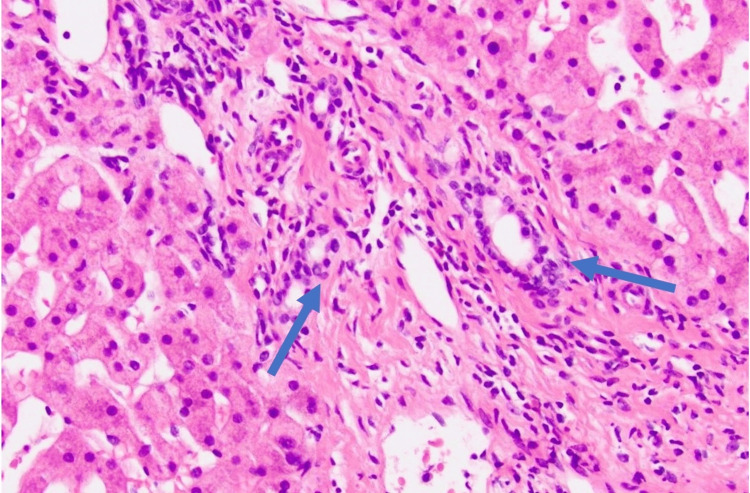
Liver biopsy revealing lymphoplasmacytic infiltrates, necrosis, and periportal proliferation (blue arrows). Stains: eosin and hematoxylin (magnification: 40x).

**Figure 2:**
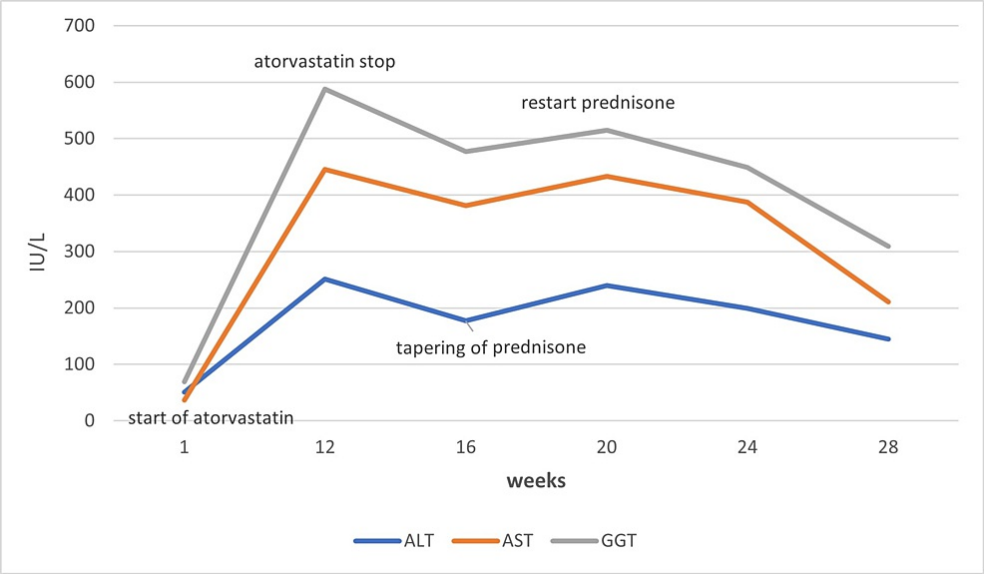
Pattern of liver enzymes after and withdrawal of atorvastatin in our patient with autoimmune hepatitis. ALT: alanine aminotransferase, AST: aspartate aminotransferase, GGT: gamma-glutamyl transferase.

Her symptoms improved, and liver chemistries showed a declining pattern. However, her visit after completion of tapering the prednisone dose revealed again markedly elevated aminotransferase levels. She was scheduled for a liver biopsy, and the finding was consistent with AIH, such as lymphoplasmacytic infiltrates, necrosis, and periportal proliferation (Figure 1). She was diagnosed with statin-induced AIH with a score of 13. She was discharged on prednisone 40 mg daily with regular follow-up for an extended glucocorticoid taper. In her recent follow-up three months later, she was asymptomatic with almost normal liver function tests (Figure 2).

## Discussion

Atorvastatin, a 3-hydroxy-3-methylglutaryl coenzyme A (HMG-CoA) reductase inhibitor, is frequently recommended for preventing cardiovascular disease and as a cholesterol-lowering agent [6]. Atorvastatin has an excellent safety profile with favorable side effects. Reported side effects of statins are tabulated in Table 3 [7].

**Table 3:** Reported side effects of atorvastatin [7].

Side effects	Number	Frequency (%)
Muscle pain	37	17
Joint pain	35	16
Elevated transaminase	24	11
Headache	1	0.46
Gastrointestinal effects	35	16
Respiratory effects	7	3.2

AIH is a rare but well-documented adverse event. The number of AIH cases induced by atorvastatin is low, and only limited cases have been published [8]. We have tabulated the reported cases of AIH caused by atorvastatin in Table 4 [5,8-12].

**Table 4:** Reported cases of AIH induced by atorvastatin [5,8-12]. M: male, F: female, NR: not reported, NA: not available, AIH: autoimmune hepatitis.

Study	Age/sex	Statin	Time to symptom onset (months)	AIH score	Diagnosis on biopsy	Treatment
Tse et al. [5]	75/F	Atorvastatin	2	8	Yes	Steroids
Khan et al. [8]	57/F	Atorvastatin	3	9	Yes	Steroids
Kawasaki et al. [9]	45/M	Atorvastatin	6	18	Yes	Steroids
Hazeghi et al. [10]	52/F	Atorvastatin	1	12	Yes	Steroids
Alla et al. [11]	47/M	Atorvastatin	4	17	Yes	Steroids
Alla et al. [11]	51/M	Atorvastatin	4	15	Yes	Steroids, azathioprine
Mohamed et al. [12]	67/M	Atorvastatin	4	NR	No	Steroids

**Figure 3:**
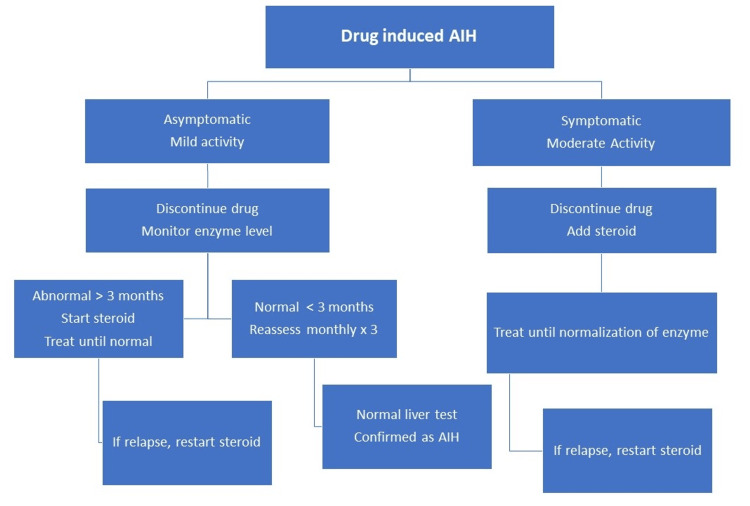
Clinical management of drug-induced autoimmune hepatitis. AIH: autoimmune hepatitis. Image credit: Zahoor Ahmed

The pathophysiology of AIH is thought to involve the binding of drug metabolites generated from the hepatic metabolism of the drug to the surface cellular proteins of hepatocytes, typically involving the phase I or phase II drug-metabolizing enzymes such as cytochrome P450 (CYP450), leading to the formation of neoantigens [13]. These neoantigens act as a trigger to induce the immune system and activation of CD8 T-cell lymphocytes, exhibiting non-selective antigen receptors, resulting in a misdirected immune response and subsequent tissue injury [9,11]. Management of drug-induced AIH involves withdrawal of the offending agent and symptomatic management, coupled with high-dose corticosteroids to reduce the inflammatory process and suppress the autoimmune response [7]. Prednisone is the most prescribed for AIH, followed by budesonide. An alternative immunosuppressant to steroids is azathioprine (Figure 3) [4,14]. Azathioprine is the treatment of choice for AIH maintenance therapy. It is generally recommended following 3-4 weeks of steroid therapy, and the recommended dose is generally 50 mg/day to minimize side effects and to anticipate liver toxicity. A combination of steroids and azathioprine is effective in maintaining remission [15].

## Conclusions

Although atorvastatin is a well-tolerated drug, drug-induced AIH is a life-threatening complication of atorvastatin therapy, which is rarely reported. Physicians should maintain a high index of suspicion for drug-induced AIH in patients presenting with hepatic dysfunction while on atorvastatin. Prompt recognition, withdrawal of the drug, and initiation of high-dose steroids are vital for favorable outcomes and for preventing long-term hepatic injury. Continued monitoring of liver function tests during follow-up visits is essential to ensure the resolution of hepatic inflammation and mitigate the risk of disease recurrence.
